# Imaging hypoxia for head and neck cancer: current status, challenges, and prospects

**DOI:** 10.7150/thno.112781

**Published:** 2025-07-11

**Authors:** Wenhui Huang, Nemin Li, Sheng Zhu, Yuze Zhang, Xinyang Song, Bin Zhang, Hao Yan, Jie Tian, Kun Wang, Shuixing Zhang

**Affiliations:** 1 State Key Laboratory of Bioactive Molecules and Druggability Assessment, the First Affiliated Hospital, Jinan University, Guangzhou, 510632, China.; 2 Guangdong Basic Research Center of Excellence for Natural Bioactive Molecules and Discovery of Innovative Drugs, College of Medicine, Jinan University, Guangzhou, 510632, China.; 3 CAS Key Laboratory of Molecular Imaging, the State Key Laboratory of Management and Control for Complex Systems, Institute of Automation, Chinese Academy of Sciences, Beijing, 100190, China.; 4 Department of Nuclear Medicine, the Affiliated Hospital of Xiangnan University, Chenzhou, 423000, China.; 5 Tsinghua Shenzhen International Graduate School, Tsinghua University, Shenzhen, 518055, China.; 6 Beijing Advanced Innovation Center for Big Data-Based Precision Medicine, School of Engineering Medicine, Beihang University, Beijing, 100191, China.

**Keywords:** tumor hypoxia, head and neck cancers, positron emission tomography, magnetic resonance imaging, optical molecular imaging

## Abstract

Hypoxia can substantially impact clinical outcomes in patients with head and neck cancer (HNC) by promoting tumor invasion, metastasis, immune escape, and therapy resistance. Given the growing interest in targeting hypoxia for cancer therapy, noninvasive methods are needed to accurately detect hypoxia and evaluate the tumor response to treatment. This review summarizes recent advances in hypoxia-targeted probes and imaging techniques, emphasizing their imaging mechanisms, strengths, and limitations. We focused on the promising clinical applications of hypoxia imaging, especially those currently used in clinics, such as positron emission tomography and magnetic resonance imaging, and highlighted their roles in guiding personalized therapy. Future directions include optimizing imaging probes to improve safety profiles, integrating multimodal imaging, applying machine learning models to analyze multiparametric data, and establishing standardized 3-dimensional *in vitro* models to better mimic hypoxia heterogeneity. These advancements are expected to considerably improve the management of patients with HNC.

## 1. Introduction

Head and neck cancer (HNC) arises from the mucosal epithelium of the oral cavity, pharynx, and larynx, and over 90% of these tumors are classified as squamous cell carcinoma 1. Existing screening strategies are known to be less effective in detecting early stages of HNCs, and physical examination remains the mainstay approach for early detection 2. Treatment for HNCs involves surgery and radiochemotherapy, depending on the disease stage and tumor type. Despite advances in diagnosis and treatment, prognostic outcomes vary among patients after treatment. Hypoxia, known to arise from an imbalance between oxygen (O_2_) demand and delivery within tumor tissues, has been identified as a key factor contributing to tumor aggressiveness and treatment resistance 3. Hypoxia is a key characteristic of solid tumors, making it a crucial target for imaging and treatment 4. Recent studies have profiled hypoxia-regulated gene expression in more than 9,000 individual tumors and have determined that HNCs exhibit some of the highest hypoxic gene expression scores 5. Extensive evidence has revealed that hypoxia affects radiotherapy outcomes by activating DNA repair mechanisms and altering cellular signaling pathways, leading to the failure of radiation-induced cytotoxicity 4, 6. Accordingly, hypoxia imaging plays a dominant role in optimizing treatment strategies and improving outcomes in patients with HNC.

Advances in imaging technology have enabled the detection and visualization of tumor hypoxia. Positron emission tomography (PET)/computed tomography (CT) imaging with hypoxia-targeted radiotracers such as [^18^F] fluoroazomycin arabinoside (^18^F-FAZA) and [^18^F] flortanidazole (^18^F-HX4) has shown promise for spatially mapping hypoxic regions in tumors 7. Magnetic resonance imaging (MRI) with or without oxygen-sensitive contrast agents offers a high spatial resolution. Additionally, optical molecular imaging (OMI) modalities, including fluorescence, phosphorescence, photoacoustic, and Cherenkov imaging, are being explored for the real-time detection of cyclic hypoxia 8. In addition to the considerable progress in imaging techniques, advances in bioimaging technology have further improved the targeting efficiency 9. With the growing interest in exploiting tumor hypoxia as a therapeutic target 10,11, hypoxia imaging has broad application prospects, such as guiding radiotherapy or immunotherapy, tracking tumor metastases* in vivo*, and screening hypoxia-activated prodrugs (HAPs) 12. This review provides a concise overview of the design strategies for hypoxia probes, with a primary focus on advances in imaging technologies and their promising applications in patients with HNC (**Graphical Abstract**).

## 2. Design Strategies for Hypoxia-Targeted Probes

The development of functional molecules and nanoparticles for hypoxia imaging enables *in vivo* monitoring of biological processes and disease states. Typically, when oxygen levels fall below 3%, the expression of hypoxia-induced factor-1alpha (HIF-1α) is upregulated, subsequently activating downstream signaling pathways critical for cellular survival. This process drives the production of hypoxia-associated enzymes and metabolites. Therefore, design strategies for hypoxia probes focus on detecting hypoxia-related analytes, which are categorized into physical, biological, and chemical subtypes (**Table 1**).

Direct measurement of partial oxygen pressure (*p*O_2_) is the most straightforward approach for assessing tumor hypoxia. Invasive oxygen microelectrodes allow clinicians to measure localized tissue oxygen concentrations at specific time points, with *in vivo* noninvasive oxygen sensors increasingly favored. These sensors often utilize phosphorescent metal complexes with long-lived emissions in which oxygen molecules quench the luminescence 13,14. Although this oxygen-induced quenching effect enables the real-time mapping of cyclic hypoxia, traditional phosphorescence-based hypoxia probes are limited to the visible and first near-infrared (NIR) wavelength regions (NIR-I, 700‒900 nm), resulting in high light scattering and low penetration depth. Recent advancements have focused on developing novel organometallic complex nanoparticles for second NIR wavelength (NIR-II, 1000‒1700 nm) imaging to improve the signal-to-noise ratio (SNR) and enhance tumor accumulation 15. However, poor biocompatibility and limited tissue penetration remain the greatest obstacles to their clinical application.

Reductive species such as nitroreductases (NTRs) and azoreductases (AzoRs) accumulate in the hypoxic tumor microenvironment 16. Hypoxia-sensitive probes that leverage nitroaromatic or azobenzene derivatives have been developed to detect redox dynamics 16-20. These moieties, which are widely used in optical and radionuclide imaging, undergo reduction processes that directly reflect the severity of hypoxia. In addition to redox-active molecules, hypoxia-induced upregulation of carbonic anhydrase IX (CAIX), a component of the complex response of cancer cells to an evolving low-oxygen environment, is a promising target for tumor hypoxia 21. Probes targeting CAIX typically combine ligand-receptor binding domains with signal-generating groups, offering a one-step synthesis and easy accessibility 22. Such strategies are commonly used in preclinical and clinical studies to provide a stable and indirect method for identifying hypoxic conditions and have considerable potential for clinical translation 23.

Hypoxia-driven anaerobic glycolysis in tumors generates lactic acid, leading to extracellular acidosis (pH 6.5‒6.9). To improve the accuracy of hypoxia detection, dual-sensing probes have been engineered to concurrently measure *p*O_2_ and extracellular pH 24. Chemical analytes linked to hypoxic signaling pathways, such as hydrogen peroxide (H_2_O_2_) 25 and hydrogen sulfide (H_2_S) 26, are emerging targets. These hypoxia-activated probes often incorporate three components: analyte-specific sensing moieties, signal transduction systems for generating detectable outputs, and bio-orthogonal functionalization for minimizing cross-reactivity. Although these probes achieve high sensitivity and rapid response, they usually require a relatively complex synthesis process, and their potential cross-reactivity with other reactive oxygen species groups compromises their specificity.

Recent progress in designing highly selective and sensitive imaging probes for tumor hypoxia has focused on the development of dual-lock fluorescent probes and dual-emissive ratiometric probes, representing a critical step toward the next generation of smart probes 27-32. Dual-lock fluorescent probes can markedly enhance detection specificity by requiring simultaneous activation with two hypoxia-associated biomarkers. These probes integrate naphthalimide fluorophores with hypoxia-sensitive moieties and dual-quenching mechanisms to achieve superior SNR, enabling noninvasive real-time monitoring of hypoxia 29,30. Dual-emissive ratiometric oxygen probes offer built-in self-calibration signal correction by integrating oxygen-sensitive fluorophores with oxygen-insensitive reference materials, enabling quantitative and reliable detection of hypoxia 31,32. This self-referencing mechanism enhances both the accuracy and reproducibility of signal acquisition, demonstrating its utility in mapping oxygen saturation (*_S_*O_2_) with spatial resolution and its compatibility with multimodal imaging platforms in preclinical studies. Moreover, advances in multimodal imaging devices have greatly facilitated the progress of hypoxia probes, with the combined advantages of different imaging modalities promising comprehensive and precise hypoxia quantification. The following sections discuss individual imaging modalities for tumor hypoxia (**Table 2**), their imaging tracers, and their applications in HNCs.

## 3. Imaging Modalities

### 3.1 PET

**Figure 1:**
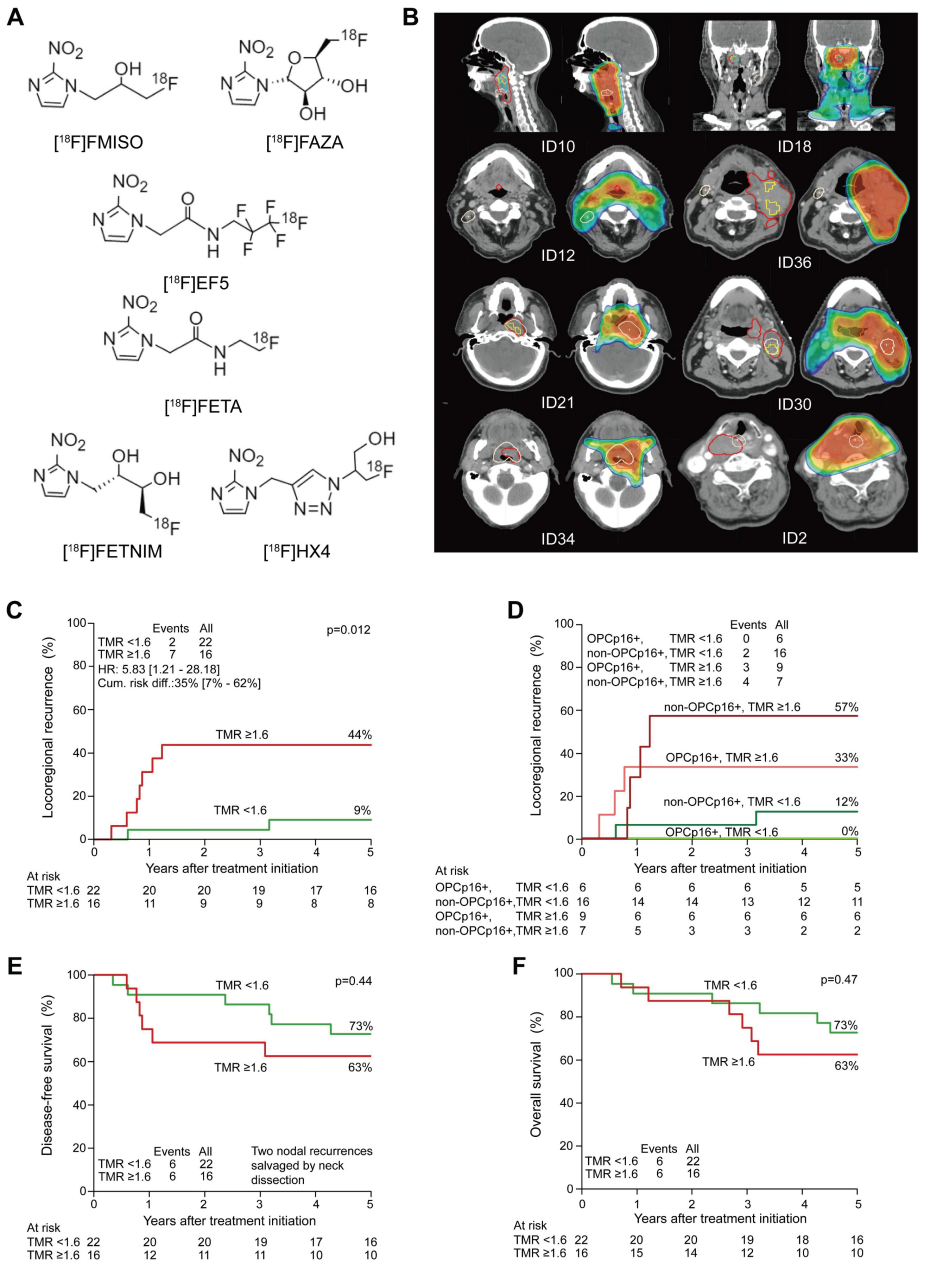
(A) Chemical structures of selected ¹⁸F-labeled 2-nitroimidazoles used in PET imaging of tumor hypoxia. Adapted with permission from 34, an open access article under a CC-BY 4.0 license, copyright 2022 Springer Nature. (B) Hypoxic subvolumes and recurrence patterns within the primary GTV (two left rows) and dose coverage (two right rows) from the radiotherapy plan. White contours and “+” symbols mark the center of recurrent tumors. Red: GTV; yellow: hypoxic subvolumes; light blue: persistent hypoxia. (C-F) Five-year outcomes comparing more vs. less hypoxic tumors. Adapted with permission from 45, copyright 2020 Elsevier. GTV: gross tumor volume; TMR: tumor-to-muscle ratio; PET: positron emission tomography.

PET is an important tool for detecting tumor hypoxia because it utilizes radiotracers that target hypoxia-specific molecules 33. These tracers, which are frequently based on the reductive tendency of nitroimidazoles, are selectively metabolized and trapped in hypoxic regions. The selection of representative ^18^F-labeled 2-nitroimidazoles for hypoxic imaging is shown in **Figure 1A** 34. [^18^F] fluoromisonidazole (^18^F-FMISO) and ^18^F-FAZA are well-investigated first- and second-generation hypoxia PET tracers used in preclinical and clinical settings 35. They exploit differences in oxygen levels between hypoxic and well-oxygenated tissues. When administered intravenously, they diffuse into cells, re-oxidize, and undergo clearance in well-oxygenated regions but are reduced to macromolecules and retained in hypoxic cells.

#### 3.1.1 First-generation radiotracer: ^18^F-FMISO

^18^F-FMISO has been widely explored for detecting hypoxia across various tumor types, demonstrating a substantial correlation between FMISO uptake and HIF-1α expression and tissue perfusion 36. ^18^F-FMISO-based PET is a promising method for quantifying hypoxia, predicting patient prognosis, and optimizing treatment planning. A meta-analysis of 323 patients with HNC from 17 different studies using hypoxic PET revealed a notable positive correlation between primary tumor volume and hypoxic tracer uptake 37. The uptake value from FMISO-based PET was identified as a prognostic indicator, indicating a worse prognosis in patients who had a high hypoxic volume on baseline images than those with a low hypoxic volume 38. In 23 patients with oral squamous cell carcinoma, the preoperative hypoxic volume, as measured on FMISO-based PET, correlated with disease-free survival and local recurrence 39. However, the results of different clinical trials remain inconsistent. For example, Löck *et al.* found weak correlations between FMISO-based PET parameters and hypoxia-associated gene expression 40. Notably, FMISO-based PET is a low-contrast imaging modality owing to its lipophilic property, with a tumor-background ratio (TBR) of 1.4‒1.6, which complicates the interpretation of PET images.

#### 3.1.2 Other nitroimidazole-based radiotracers

A new generation of hypoxia radiotracers has evolved from nitroimidazole-based compounds such as etanidazole (EF3 and EF5). Although these agents exhibit higher lipophilicity than ^18^F-FMISO, along with easy tissue penetration and short injection-to-imaging time, their slow clearance from normoxic tissues results in a suboptimal TBR 41. Second-generation tracers, such as ^18^F-FAZA, address this limitation by enhancing hydrophilicity, achieving faster clearance from non-hypoxic regions, and improving the TBR 42. Further advancements have focused on enhancing hydrophilicity to reduce nonspecific retention, generating third-generation agents with rapid renal clearance, such as ^18^F-HX4, which has shown a 6-fold and 3-fold better TBR than ^18^F-FMISO and ^18^F-FAZA, respectively 43. Currently, ^18^F-FAZA and ^18^F-HX4 are being extensively explored in clinical studies. In a study involving 23 patients with HNC, FAZA uptake correlated with disease progression 44. A prospective study involving 38 patients with non-metastatic HNC (**Figure 1B-F**) revealed that higher uptake on FAZA-based PET correlated with a higher risk of local recurrence after an 8-year follow-up period 45. For ^18^F-HX4, a multicenter study confirmed the reproducibility in hypoxia quantification, with scan parameters revealing robust intersession correlations 46. Additionally, HX4 uptake was substantially correlated with pimonidazole and CAIX expression, validating its biological relevance 47. These advancements highlight the translational potential of next-generation radiotracers for refining hypoxia-driven therapeutic strategies for HNC.

Although emerging evidence underscores the clinical potential of hypoxia-targeted radiotracers, notable challenges remain to be addressed. PET imaging, despite its functionality, is constrained by its limited spatial resolution (5‒7 mm) and inherently low soft-tissue contrast. Tumor hypoxia exhibits spatial heterogeneity and dynamic temporal evolution, making it difficult to accurately capture hypoxic regions using a single PET scan 48. This is particularly relevant in hypoxia image-guided radiotherapy, in which hypoxic regions need to be precisely delineated. Furthermore, conventional hypoxic radiotracers, such as ^18^F-FMISO, suffer from prolonged uptake kinetics, necessitating delayed imaging (120-180 min post-administration), resulting in a suboptimal SNR. Advanced pharmacokinetic modeling techniques are leveraged to differentiate true hypoxia-specific tracer uptake from nonspecific background signals, thereby improving quantitative accuracy.

### 3.2 MRI

MRI utilizes robust magnetic fields and radiofrequency pulses to generate high-resolution functional images 49. MRI primarily employs two contrasting mechanisms to assess hypoxia. One method uses paramagnetic contrast agents that shorten T1 relaxation times and enhance the signal intensity on T1-weighted images. The other relies on oxygen-sensitive imaging sequences such as blood oxygen level-dependent (BOLD) and oxygen-enhanced MRI, which detect endogenous signal variations linked to tissue oxygenation levels. In the subsequent sub-sections, we review the principles, clinical advantages, and limitations of each method in the context of hypoxia imaging.

#### 3.2.1 BOLD-MRI

Paramagnetic deoxyhemoglobin increases the transverse relaxation rate (R2*, defined as 1/T2*) of water protons in blood and tumor tissues, forming the basis for oxygenation-sensitive imaging via BOLD-MRI 50. Tumor R2* decreases with elevated blood *_S_*O_2_ levels during hyperoxic gas inhalation, enabling BOLD sequences to detect oxygenation changes with high sensitivity. However, the relationship between R2* and direct tissue *p*O_2_ measurements remains controversial. Preclinical studies have reported statistically significant associations between tumor R2* and tissue *p*O_2_ levels 51, whereas clinical trials, including a 10-patient cohort analysis, failed to detect a significant correlation 52. Although R2* values were found to positively correlate with HIF-1α expression or pimonidazole staining in several studies 53, the observed signal changes were subtle and markedly susceptible to O_2_ changes in large vessels and surrounding tissues, hindering their ability to detect regional hypoxia. Furthermore, BOLD imaging sequences are inherently sensitive to motion artifacts and magnetic susceptibility at air-tissue interfaces, complicating their application in HNC. These technical and biological constraints underscore the need for complementary methods to assess hypoxia and validate the BOLD-derived findings in clinical settings.

#### 3.2.2 Oxygen-enhanced MRI (OE-MRI)

OE-MRI is an emerging technique that measures free oxygen in plasma and interstitial fluid to evaluate tissue oxygenation 54. Molecular oxygen is paramagnetic, and the longitudinal relaxation rate (R1 = 1/T1) increases linearly with increasing O_2_ concentrations. In well-oxygenated tumors, elevated dissolved O_2_ shortens T1, resulting in positive proton signal changes (ΔR1) similar to those observed in healthy tissues. Positive ΔR1 indicates efficient O_2_ delivery, while negative ΔR1 identifies hypoxic regions. OE-MRI provides direct insights into tumor hypoxia and has garnered considerable research interest. Notably, O'Connor *et al.* introduced the concept of "perfused Oxy-R,” regions with intact perfusion but absent oxygen enhancement, to distinguish hypoxic from well-oxygenated regions in preclinical models 55. A subsequent first-in-human study demonstrated the feasibility and repeatability of OE-MRI, revealing that perfused Oxy-R could stratify patients based on post-radiotherapy hypoxia modulation, highlighting its utility in monitoring treatment responses and identifying non-responders 56. Recently, OE-MRI was successfully transformed into volumetric and dynamic hypoxia imaging on an MRI and linear accelerator (MR-Linac) in a cohort of 15 healthy participants and 14 patients with HNC 57, achieving a 98% success rate (50/51 scans), thereby indicating its potential for clinical use in hypoxia image-guided radiotherapy.

Likewise, OE-MRI is subject to translational challenges. Key challenges include obtaining optimal acquisition timing and susceptibility artifacts at air-tissue interfaces 58,59. Standardizing MRI protocols is critical for accurate ΔR1 quantification. Additionally, advanced computational methods such as data-driven clustering algorithms may improve hypoxia segmentation and artifact correction. Addressing these barriers is crucial for advancing OE-MRI from experimental validation to routine clinical workflows.

#### 3.2.3 Dynamic contrast-enhanced MRI (DCE-MRI)

In tumors, hypoxic regions exhibit reduced blood flow and vascular permeability, resulting in slower clearance of contrast agents than well-oxygenated regions. DCE-MRI uses rapid sequential imaging to track the kinetics of gadolinium-based contrast agents, thereby providing indirect insights into tissue perfusion and oxygen delivery. By utilizing tracer kinetic modeling of serial MRI acquisitions, DCE-MRI quantifies microvascular parameters such as the volume transfer constant (*K*^trans^), reflux rate (*K*_ep_), and extracellular volume fraction (*V*_e_). Although early studies reported weak correlations between these parameters and direct hypoxia markers, such as pimonidazole staining 60, Gaustad *et al.* demonstrated that *K*^trans^, derived from pharmacokinetic modeling incorporating arterial input functions, showed a substantial correlation with tumor hypoxia in animal models 61. DCE-MRI parameters have shown utility in detecting baseline and treatment-induced hypoxia, as well as in predicting hypoxia-induced radiation response and metastatic potential. However, it is crucial to note that DCE-MRI does not directly measure O_2_ levels, necessitating integration with additional imaging modalities or molecular biomarkers for a comprehensive assessment 62. Future efforts should focus on standardizing acquisition protocols, open data sharing, and large-scale multicenter validation studies to establish robust hypoxia-specific imaging biomarkers.

In summary, PET and MRI have demonstrated promising clinical results for detecting tumor hypoxia. However, their widespread application is constrained by high costs, time-consuming scans, and reliance on ionizing radiation or strong magnetic fields. These unmet clinical requirements have led to advancements in OMI.

## 4. OMI

**Figure 2:**
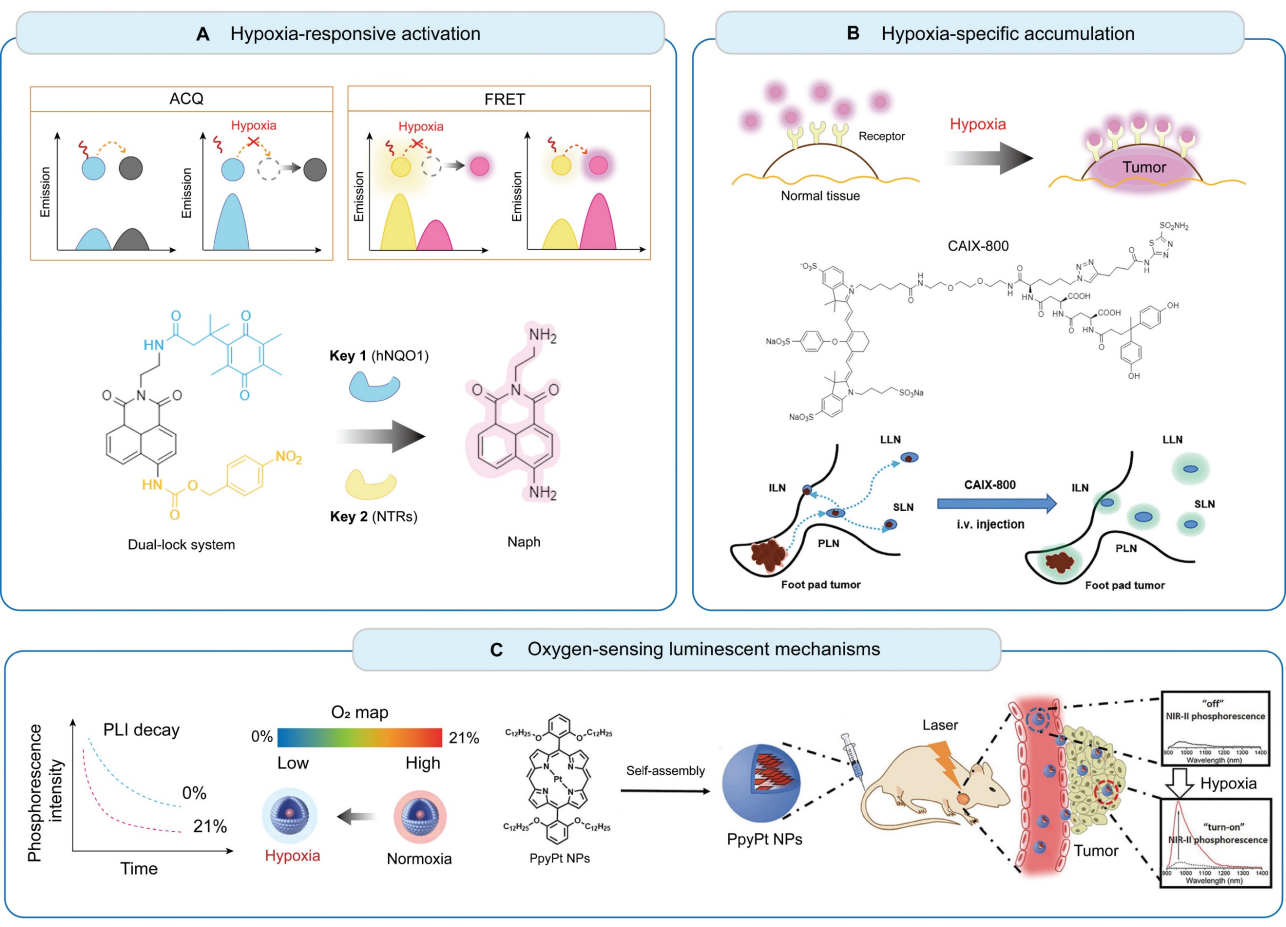
Illustration of the molecular mechanisms and the bioimaging applications of fluorescent hypoxia probes. (A) Hypoxia-responsive activation utilizing FRET and ACQ for enhanced SNR. (B) Hypoxia-specific accumulation targeting hypoxia-induced enzymes or proteins. Adapted with permission from 106, copyright 2022 Springer Nature. (C) Oxygen-sensing luminescent mechanisms based on oxygen-sensitive metal complexes with long-lived emissions. Adapted with permission from 13, copyright 2022 Wiley. FRET: fluorescence resonance energy transfer; ACQ: aggregation-caused quenching; NTRs: nitroreductases; AzoRs: azoreductases; CAIX: carbonic anhydrase IX; SNR: signal-to-noise ratio; PLN, popliteal lymph node; SLN, sciatic lymph node; ILN, inguinal lymph node; LLN, lumbar lymph node.

Optical imaging enables the visualization and quantification of biological processes at the molecular level, offers high practicality and cost-friendliness, and is emerging as a fundamental tool for imaging tumor hypoxia in the field of basic science 8. Diverse optical techniques, including fluorescence, phosphorescence, photoacoustic, and Cherenkov imaging, employ hypoxia-specific probes and advanced imaging systems to visualize and quantify hypoxic regions within tumors (**Figure 2**).

### 4.1 Fluorescence imaging

Fluorescence imaging is considered the most sensitive and widely used optical method for detecting hypoxia using two typical strategies: hypoxia-responsive activation and hypoxia-specific accumulation 63.

#### 4.1.1 Hypoxia-responsive activation

These fluorescent probes function as O_2_ sensors, enabling stimuli-specific "off-on" activation to enhance SNR within hypoxic regions. They typically contain hypoxia-responsive enzymes or molecules that modulate fluorescence intensity or spectral characteristics in response to hypoxic regions. Two common methods used to design such probes include aggregation-caused quenching (ACQ) and fluorescence resonance energy transfer (FRET) 64. ACQ occurs when fluorophores in close proximity undergo energy transfer, leading to fluorescence quenching. When encapsulated tightly within nanocarriers equipped with reductive enzymes, such as azobenzene derivatives 65 or nitroreductases 66, fluorescence is selectively activated in hypoxic regions owing to carrier disassembly. The coupled FRET pairs are linked via hypoxia-cleavable groups. Under low-oxygen conditions, FRET occurs when a donor fluorophore transfers energy to a receptor fluorophore through non-radiative mechanisms, furnishing a direct response to targeted analytes with improved specificity and SNR 67. Although ACQ and FRET probes are well-established in preclinical imaging, their applications in patients with HNC remain unexplored.

#### 4.1.2 Hypoxia-specific accumulation

Hypoxia-specific probes selectively bind to specific biomarkers that are upregulated in hypoxic regions. These probes typically target HIF-1α or its downstream regulators, such as CAIX, glucose transporter-1, vascular endothelial growth factor, and reductive enzymes, including nitroimidazole, nitrobenzyl alcohol, and azobenzene derivatives, which are commonly utilized in both nanoprobes and molecular probes. CAIX is a transmembrane protein overexpressed in hypoxic cells and serves as a prognostic factor in patients with HNC 68. CAIX is highly expressed in primary and metastatic lesions within several tumor types and is strongly correlated with reduced oxygenation status 69. Numerous CAIX-targeted antibodies or small-molecule inhibitors have been developed and labeled with fluorophores or radionuclides 22,70. In a previous study, we synthesized CAIX-800, a dual-motif CAIX ligand conjugated to IRDye 800CW, and demonstrated its targeting efficiency in orthotopic and metastatic NPC models 71. Future studies must focus on biocompatibility, minimal toxicity, and improved tissue penetration to advance the application of these probes toward clinical trials.

### 4.2 Phosphorescence lifetime imaging (PLI)

PLI utilizes oxygen-sensitive phosphorescent molecules to directly quantify O_2_ levels using time-resolved cameras. Phosphorescent transition-metal complexes are typical O_2_ sensors, including ruthenium, iridium, and metalloporphyrin complexes, which exhibit reversible and linear responses to changes in O_2_ concentration 72. Early-generation probes demonstrate poor water solubility, ultraviolet-visible emission-induced tissue scattering, and limited penetration depth, rendering them unsuitable for *in vivo* studies. Ideally, phosphorescent sensors should exhibit water solubility, low toxicity, high photostability, and NIR emission for deep tissue imaging. Zheng *et al.* developed Ir-PVP, a water-soluble NIR-emitting nanoprobe that enabled the detection of solid tumors and metastatic cancer cells with high sensitivity and SNR 73. The authors further developed a ratiometric hypoxia imaging nanoprobe by combining semiconducting polymers with phosphorescent phosphors, demonstrating better photostability and lower toxicity than early probes 74. This probe enabled the quantification of reversible hypoxia (1.56‒2.64 mmHg) during radiotherapy. Their findings indicated that the tumor reoxygenation efficiency during the first radiation fraction critically predicted treatment outcomes, highlighting the potential of PLI for optimizing radiation treatment planning. Although PLI offers radiation-free, quantitative, and dynamic hypoxia assessments at a low cost, this technique requires specialized equipment, including lasers and time-resolved cameras, which are complex to set up and operate. A complex imaging system and limited penetration depth may limit its accessibility in clinical settings.

### 4.3 Photoacoustic imaging (PAI)

PAI employs laser-induced photoacoustic effects and ultrasound detection to visualize biological events at centimeter-level penetration depths. This technique quantifies oxygen levels and vascular networks by detecting variations in the absorption spectra of endogenous chromophores (such as hemoglobin and melanin) or exogenous contrast agents 75. Label-free PAI measures oxygenated and deoxygenated hemoglobin concentrations through spectral unmixing and assesses tumor hypoxia based on *_S_*O_2_ and blood perfusion, which is similar to the principle of BOLD-MRI. This technique offers cost-effective, high-resolution imaging 76-78. For instance, Ron *et al.* developed a volumetric multispectral optoacoustic tomography system to dynamically capture tumor oxygenation kinetics, distinguishing between normoxic rims, normoxic cores, and hypoxic cores. This novel system sensitively detects cyclic hypoxia, demonstrating potential for therapeutic monitoring 79. Rich *et al.* used PAI to track tumor oxygenation changes in human papillomavirus (HPV)-positive and -negative xenografts during radiotherapy, indicating the utility of PAI as a radiation response biomarker in patients with HNC 78. In addition to label-free PAI, advanced techniques utilize exogenous agents to enhance the detection of hypoxia. Tomaszewski *et al.* introduced oxygen-enhanced optoacoustic tomography (OE-OT) and DCE-OT, which revealed strong correlations between *in vivo* imaging parameters (Δ*_S_*O_2_ and ΔICG) and *ex vivo* hypoxia quantification 80. Despite numerous optoacoustic probes that have improved imaging quality, their safety profiles and long-term biocompatibility remain elusive 81,82.

### 4.4 Cherenkov luminescence imaging (CLI)

**Figure 3:**
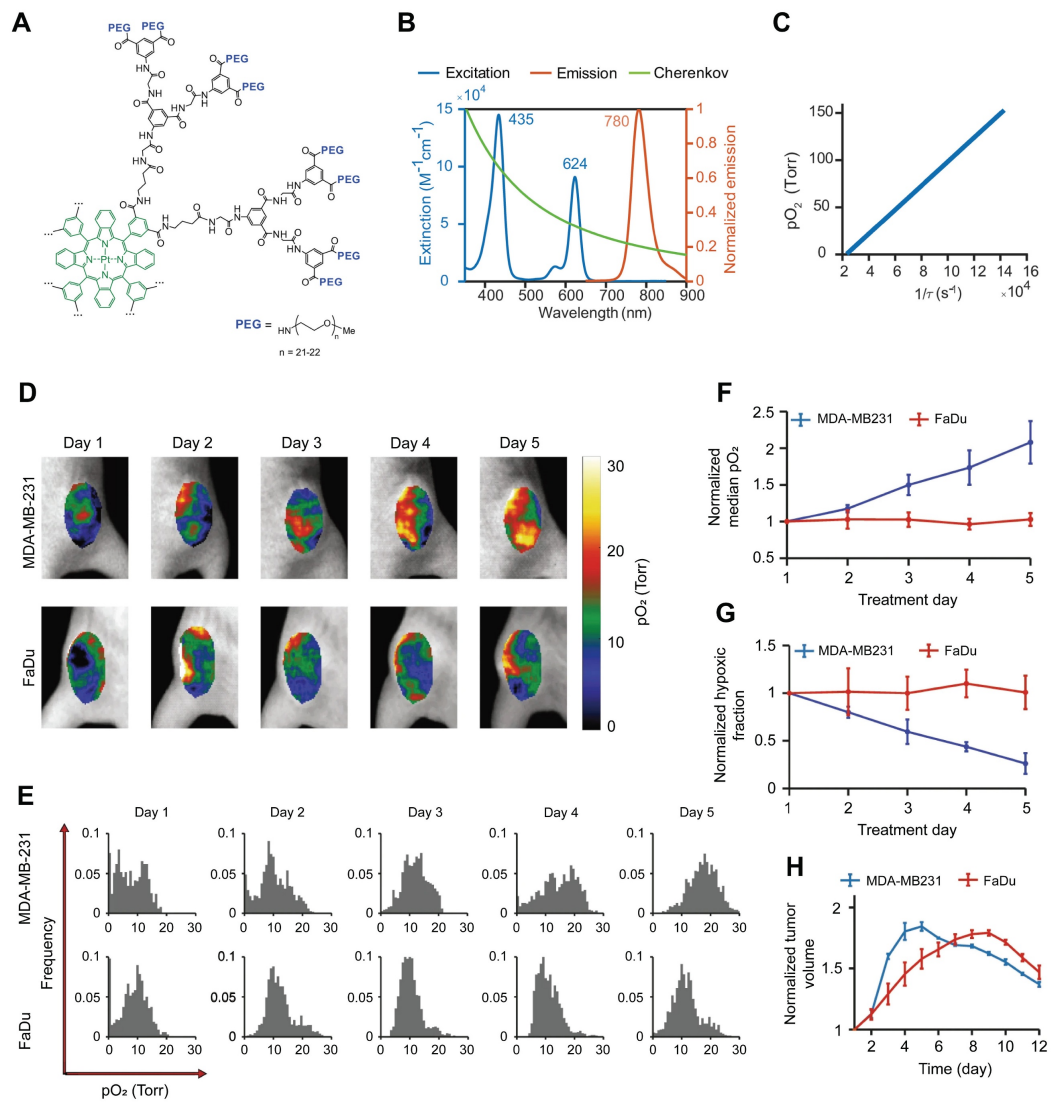
(A) Chemical structure of the phosphorescent oxygen probe Oxyphor PtG4. (B) Excitation and emission spectra of Oxyphor PtG4 and the spectrum of Cherenkov radiation. (C) Oxygen quenching calibration curve of Oxyphor PtG4. (D-E) *In vivo* longitudinal *p*O_2_ imaging of two tumor models over 5 days of fractionated radiotherapy. (F-G) Quantitative analysis of median *p*O_2_ levels and hypoxic fraction changes during treatment. (H) Relative tumor volume changes normalized to baseline prior to radiotherapy. Adapted with permission from 85, an open access article under a CC-BY 4.0 license, copyright 2020 Springer Nature.

CLI is a unique optical imaging technique that captures visible light photons generated when high-energy radiation beams traverse biological tissues. The broadband spectrum of Cherenkov light enables noninvasive quantification of blood oxygenation through the spectral unmixing of oxygenated and deoxygenated hemoglobin absorption profiles. Zhang *et al.* demonstrated a linear correlation between the Cherenkov light intensity and tissue *_S_*O_2_, facilitating real-time oxygenation monitoring during radiotherapy 83. Although *_S_*O_2_ serves as an indirect marker of hypoxia, its relationship with tissue *p*O_2_ remains unclear. Direct *p*O_2_ quantification can be achieved by employing phosphorescent oxygen sensors. Recently, Cherenkov-excited luminescence imaging has been used to quantify tumor hypoxia and its changes during radiation therapy 84-87. For instance, Pogue *et al.* developed a PtG4 probe that showed preferential accumulation and retention within tumors (>5 days) in breast and HNC xenografts 85 (**Figure 3**). By measuring phosphorescence lifetimes during fractionated radiotherapy (5 Gy per fraction), the authors demonstrated the potential for mapping tumor oxygenation *in vivo* with submillimeter resolution, revealing heterogeneous hypoxic responses across treatment fractions.

Overall, CLI demonstrates considerable potential for assessing therapeutic responses. Combined with oxygen sensors, CLI offers real-time spatial mapping of tissue *p*O_2_ with a submillimeter resolution (~1 mm) for surface imaging. However, two limitations currently limit its clinical application. First, CLI typically provides two-dimensional (2D) images, which hampers accurate signal intensity quantification. Second, it has limited tissue penetration and low sensitivity to deep targets. Future research should prioritize the implementation of 3D tomographic reconstruction techniques and improve oxygen sensors to enhance specificity, sensitivity, biocompatibility, and SNR.

## 5. Promising Application of Imaging Hypoxia in HNC

With the availability of diverse imaging modalities and innovative probes, hypoxia imaging has demonstrated promising potential for managing patients with HNC. Despite substantial evidence on the feasibility and efficiency of hypoxia-targeted strategies, substantial gaps remain in translating these discoveries into clinically validated techniques. A major obstacle in the clinical trials exploring hypoxia-targeted strategies is the lack of reliable information on tumor hypoxia; thus, there is a pressing need for clinically applicable methodologies that may enable quantification, mapping, and monitoring of hypoxia. We systematically reviewed all relevant clinical trials registered at ClinicalTrials.gov over the past decade (**Table 3**). Most clinical trials have focused on assessing the efficacy of PET-based hypoxia-selective tracers. Given that hypoxia-specific PET imaging is associated with inherent weaknesses, the potential of other methodologies, including MRI and PAI techniques that quantify blood flow or oxygenation-dependent changes, is being explored. While these trials have primarily focused on risk stratification, hypoxia-guided dose escalation, and monitoring of therapeutic response, emerging studies highlight transformative applications in basic science and clinical management of HNC. Next, we elaborate on four promising directions, underscoring the expanding role of hypoxia imaging in precision oncology.

### 5.1 Guiding radiotherapy: a pending need for convincing evidence

**Figure 4:**
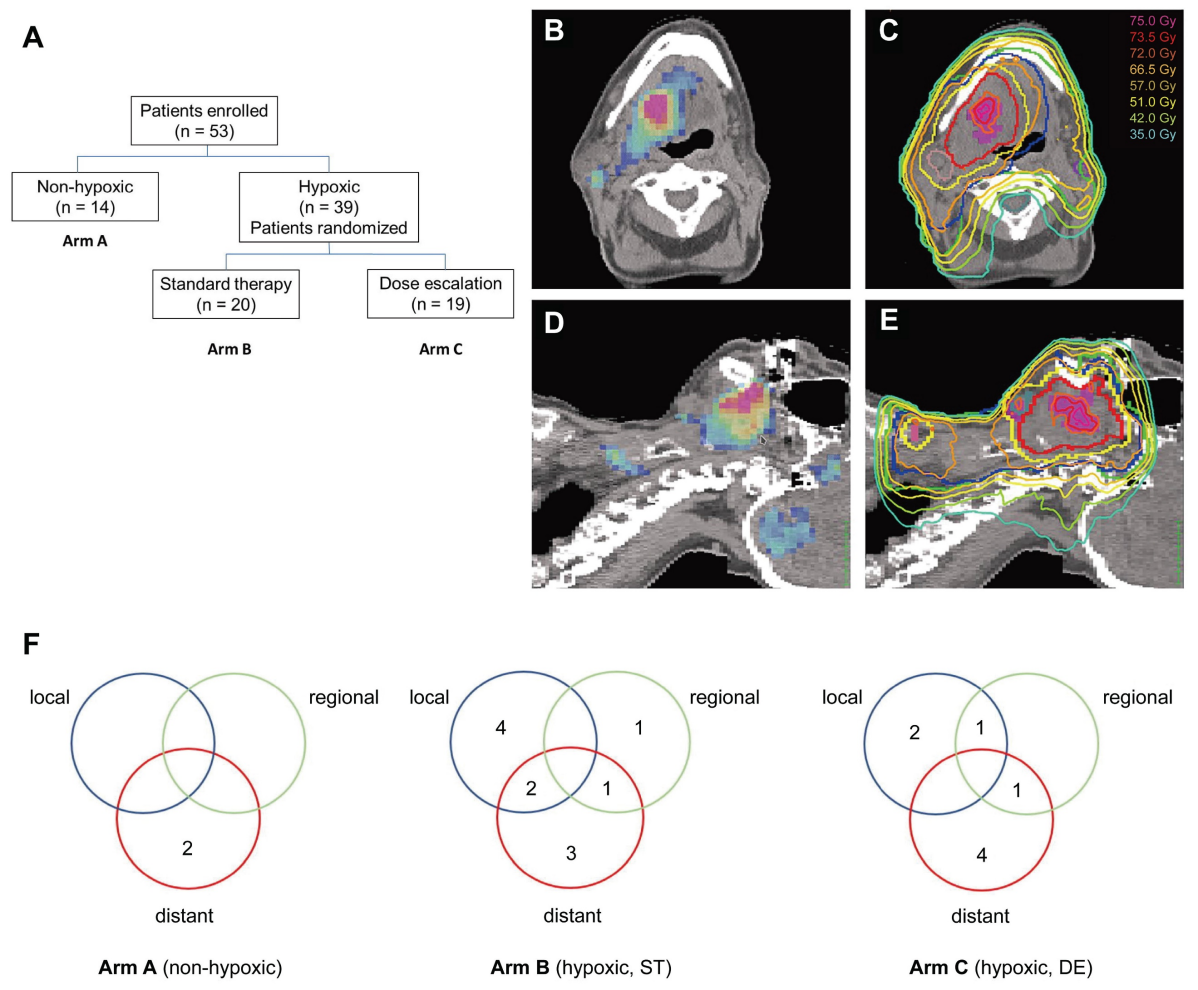
(A) Flowchart of a randomized phase II trial using ^18^F-FMISO-based PET/CT. (B-E) FMISO PET images (B, D) and corresponding planning CT (C, E) illustrating a dynamic dose painting strategy based on hypoxic subvolumes. (F) Recurrence patterns across treatment arms A, B, and C. Adapted with permission from 90, copyright 2022 Elsevier. PET: positron emission tomography; CT: computed tomography.

Hypoxic tumor cells exhibit 2‒3-fold greater radio-resistance than normoxic cells, contributing to treatment failure and recurrence 12. Modern radiotherapy techniques, such as intensity-modulated radiation therapy, enable precise dose escalation to subregions within tumors 88. This has sparked interest in hypoxia-guided dose painting, which allows selective dose escalation within radioresistant regions while sparing adjacent normal tissues. Numerous clinical trials have attempted to validate the feasibility and effectiveness of this strategy 89-95. For example, a randomized phase II trial (n = 25) assessed the feasibility, toxicity, and efficacy of hypoxia-guided dose escalation 89. All patients who underwent baseline FMISO PET/CT were randomized to receive standard chemoradiation (70 Gy/35 fractions) or hypoxic volume dose escalation (77 Gy/35 fractions). After a follow-up of 27 months, locoregional control in patients receiving standard radiation was markedly worse than that in those undergoing dose escalation (44% vs. 100%). Although dose escalation to hypoxic volumes was feasible without increasing toxicity, a subsequent trial (n = 53) failed to detect a significant difference (*P* = 0.150) in the 5-year locoregional control between the escalated (16/19) and standard (10/17) groups 90 (**Figure 4**). A phase II trial (N = 152) evaluated the feasibility of hypoxia-guided de-escalated chemoradiotherapy using ¹⁸F-FMISO PET. Patients with non-hypoxic tumors (n = 128) received 30 Gy of chemotherapy, whereas those with hypoxic tumors (n = 24) received standard chemoradiotherapy at a dose of 70 Gy. The 30-Gy cohort achieved a 2-year locoregional control rate of 94.7% and a 2-year overall survival of 100%, with significantly reduced acute grade 3‒4 toxicity (32% vs. 58.3%; *P* = 0.02) compared with the standard 70-Gy cohort. Based on these findings, hypoxia imaging helped to stratify patients for de-escalated therapy, thereby reducing toxicity while preserving efficacy 91. The DAHANCA 24 trial prospectively evaluated FAZA PET/CT-defined hypoxia and confirmed its role in the prognostic stratification of patients with non-metastatic HNC (n = 38) undergoing radiotherapy 45. At a median follow-up of 7.8 years, baseline hypoxia was found to be associated with a 5.8-fold increased risk of locoregional recurrence (44% vs. 9%; hazard ratio [HR] = 5.8), particularly in HPV-negative tumors (57% recurrence). Spatial analysis revealed that recurrence overlapped with baseline hypoxic subvolumes in 2 out of 6 hypoxic cases, supporting the feasibility and efficiency of FAZA PET/CT for guiding treatment intensification. Taken together, while hypoxia-guided dose painting is feasible and well-tolerated, large-scale multicenter trials with extended follow-up periods are necessary to assess its safety and efficacy, optimize patient selection, and standardize hypoxic subvolume delineation protocols.

### 5.2 Guiding immunotherapy: an emerging frontier in clinical oncology

**Figure 5:**
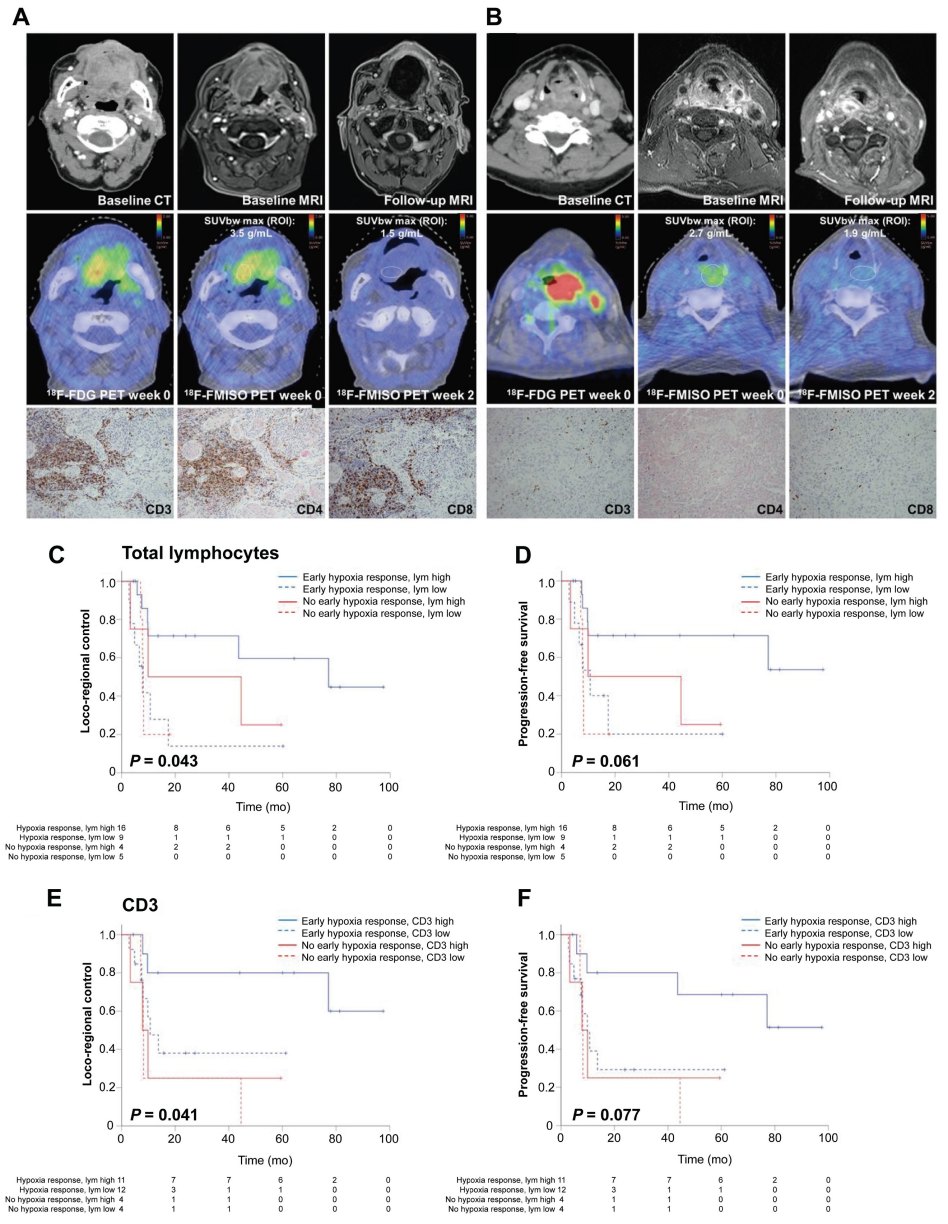
(A-B) Representative patients with HNSCC exhibiting high (A) vs. low (B) intratumoral lymphocyte levels under hypoxic conditions. (C-F) Kaplan-Meier curves showing that early hypoxia resolution and high tumor-infiltrating lymphocyte levels correlate with better outcomes. Adapted with permission from 99, copyright 2021 SNMMI. HNSCC: head and neck squamous cell carcinoma.

Hypoxic tumors promote immunosuppression by recruiting tumor-associated macrophages and inhibiting cytotoxic T-cell activity 96. Recent findings have identified an association between lymphocyte infiltration and hypoxia, suggesting the potential for hypoxia image-guided immunotherapy 97. In preclinical models, Reeves *et al.* demonstrated that FMISO-based PET could identify immunosuppressive hypoxic regions, enabling the precise timing of evofosfamide in tumors refractory to programmed cell death protein 1 and cytotoxic T-lymphocyte-associated protein 4 inhibitors 98. A clinical trial involving 49 patients with HNC demonstrated that an early hypoxic response (assessed via FMISO-based PET) and high tumor-infiltrating lymphocyte levels correlated with improved treatment outcomes 99 (**Figure 5**). The authors successfully established a hypoxia-immunity prognostic scoring system that enables the prediction of treatment responses and outcomes in patients undergoing chemoradiotherapy. Another clinical trial systematically monitored hypoxia evolution using FMISO-based PET imaging at weeks 0, 2, and 5 of the therapy 100. Patients with persistent hypoxia and high programmed cell death ligand 1 (PD-L1) expression exhibited unfavorable outcomes, whereas those with an early hypoxia response achieved favorable survival regardless of PD-L1 levels. This suggests that combining hypoxia-targeted therapies, immune checkpoint inhibitors, and hypoxia-guided dose escalation could enhance the therapeutic efficacy. Accordingly, hypoxia imaging may stratify patients who would benefit from combined immunotherapy-radiotherapy regimens, although validation in large-scale randomized trials remains critical.

### 5.3 Tracking metastases: potential for early detection

Current imaging techniques are unable to detect most metastases at an early stage. Tumor hypoxia, a critical driver of metastatic progression, influences multiple steps in the metastatic cascade 101. Given the genomic consistency between primary tumors and metastases 102, hypoxia may be an attractive target for detecting early metastases. In a cohort of 45 patients with HNC, Bandurska-Luque *et al.* reported a significant but weak correlation between FMISO-based PET hypoxia in primary tumors and metastatic lymph nodes (r = 0.36; *P* = 0.015) 103. In a cohort study of 281 patients with HNC undergoing chemoradiotherapy, persistent hypoxia on FMISO-based PET during treatment was associated with a 3.5-fold increased risk of organ metastasis (HR = 3.51; *P* = 0.04) and worse overall survival (HR = 2.66;* P* = 0.02). None of the patients with baseline hypoxia-negative tumors experienced organ metastasis 104. Although hypoxia probes have demonstrated satisfactory performance in tumor detection, their application in detecting metastases remains underexplored. Given the established role of CAIX as a promising biomarker of metastatic progression 105, our group developed a CAIX-targeted probe (CAIX-800) and investigated its targeting efficiency in preclinical models, revealing its superior capability to capture clinically undetectable lymph node metastases (< 5 mm) 106. Accordingly, hypoxia-specific probes, such as CAIX-800, could be promising for detecting micrometastases.

### 5.4 Screening HAPs: a new frontier in oncology

HAPs selectively release cytotoxic agents in hypoxic tumors with improved therapeutic efficiency 107. Two HAPs, tirapazamine and evofosfamide, have demonstrated clinical potential despite failing to achieve regulatory approval. The traditional paradigm for assessing the efficacy of HAPs relies on invasive pre- and post-treatment biopsies, underscoring the need for noninvasive hypoxia imaging to monitor the treatment response and evaluate pharmacodynamics 108. In phase I/II trials involving 44 p16-negative patients with HNC, the combination of tirapazamine plus cisplatin and radiotherapy was assessed using ^18^F-FMISO-based PET with a 7-year median follow-up, demonstrating a substantial benefit of tirapazamine in locoregional control and progression-free survival in 35 patients with hypoxic tumors 109. A similar trial using ^18^F-FAZA-based PET for hypoxia assessment in 41 patients with HPV-negative HNCs confirmed improved locoregional control and failure-free survival with tirapazamine in patients with hypoxic tumors 42. Furthermore, hypoxia imaging can delineate regions of persistent hypoxia after HAP treatment, indicating drug-resistant niches. Hypoxia imaging may accelerate HAP development and aid in identifying promising candidates for clinical evaluation.

## 6. Challenges and Future Directions

### 6.1 Current challenges

While PET/CT remains the dominant imaging modality for hypoxia assessment in patients with HNC, most studies primarily utilize the first-generation hypoxia PET tracer (^18^F-FMISO), which holds clinical promise but exhibits slow tumor diffusion kinetics, suboptimal SNR, and limited spatial resolution 110,111. Although MRI-based modalities, including OE-MRI, BOLD-MRI, and DCE-MRI, are underexplored in clinical trials, they represent emerging alternatives that leverage endogenous contrast mechanisms without exogenous tracers 112,113. Notable challenges include technical standardization (e.g., variable oxygen delivery protocols), magnetic susceptibility artifacts at air-tissue interfaces (e.g., oral cavity and sinuses), and the absence of universally accepted MRI-derived hypoxia parameters that correlate with histopathological standards. These drawbacks should be addressed for clinicians to develop MRI as a clinically applicable imaging technique for tumor hypoxia 114. Despite their high sensitivity and specificity, optical imaging modalities are absent from clinical trials owing to their inherent depth limitations (1‒2 cm) 8. Moreover, the clinical translation of new imaging agents requires a long-term and strict regulatory process, which can explain the gap between basic research and clinical application.

### 6.2 Multimodal imaging techniques and advanced analytics

With the growing interest in recognizing the complex interactions between hypoxia status and cancer biology and therapy, advances in imaging that reach deeper and broader, ranging from the microscopic to the macroscopic scale, would provide new insights into a comprehensive image of tumor hypoxia and its underlying mechanisms. Multi-imaging modalities integrate the complementary strengths of diverse techniques, address the limitations mentioned above, and offer complementary, accurate, and detailed information 115. For instance, hybrid PET/MRI combines the high sensitivity of PET with the anatomical and functional resolution of MRI, facilitating the precise delineation of hypoxic regions. The integration of optoacoustic imaging and ultrasound systems enables the unique scalability of spatial resolution and depth penetration across both optical and ultrasonic dimensions, with the potential for the *in vivo* tracking of cyclic hypoxia.

Given the evolving multiparametric data from these integrated systems, analyzing data from different origins with varying temporal and spatial resolutions and formats is a challenging task that requires artificial intelligence (AI)-driven methods 116,117. A recent study developed a multi-view machine learning framework that synergizes dynamic PET and multiparametric MRI data to characterize intratumoral subregions aligned with tumor histology 116. AI-based approaches could quantify the phenotypic heterogeneity of hypoxic tumors and predict patient outcomes.

### 6.3 Advanced study models for future investigations

Current preclinical models do not adequately elucidate the hypoxic heterogeneity in human tumors. Traditional cell line-based animal models fail to reflect the complex biology of parental tumors 118. Although patient-derived xenografts, generated by transplanting patient-derived tumor samples into mice, are closer to ideal models, they are expensive and time-consuming. Tumor organoids or 3D cell culture systems derived from patient tumor tissues offer physiologically relevant platforms for examining tumor hypoxia 119,120. Culturing under controlled oxygen gradients and stromal interactions enables high-throughput screening of hypoxia-targeted agents (e.g., HAPs and imaging probes) and real-time tracking of therapeutic responses 121. Such a robust and versatile platform can enable personalized and precise imaging of tumor hypoxia.

## 7. Conclusions

Hypoxia imaging has emerged as a critical tool for managing patients with HNC, and recent advancements have considerably improved the detection and longitudinal monitoring of tumor hypoxia. Each imaging technique offers unique advantages and insights, and their integration provides a comprehensive view of tumor oxygenation and related biological processes. In addition to diagnosis, hypoxia imaging plays a pivotal role in guiding personalized treatment strategies such as hypoxia-guided radiotherapy or immunotherapy, optimization of HAP synthesis, and hypoxia-targeted delivery of immune checkpoint inhibitors.

Despite notable advances, existing hypoxia-targeted probes have several limitations, including suboptimal specificity, limited tissue penetration (especially in optical probes), potential off-target reactivity, and complex synthesis routes. Imaging devices also encounter challenges, such as low spatial resolution (PET/CT), susceptibility artifacts (MRI), and shallow imaging depth (optical techniques), which collectively impede their clinical utility. Furthermore, regulatory hurdles and the lack of standardized validation models contribute to the translational gap.

Future directions include improving probe safety profiles, simplifying synthesis routes, exploring NIR-II platforms for improved depth, and integrating multimodal imaging systems to enhance translational potential. Simultaneously, 3D preclinical models, including patient-derived tumor organoids, large-scale multicenter trials, and AI-driven analytics, will be valuable in identifying robust hypoxia imaging biomarkers. Collectively, these efforts will help overcome current barriers and maximize the potential of hypoxia imaging in precision oncology, ultimately improving therapeutic outcomes in patients with HNC.

## Figures and Tables

**Table 1:** Design strategies for hypoxia-targeted probes.

Categories	Mechanism	Representative Probes	Imaging Devices	Advantages	Disadvantages	Recent Innovations	References
Physical	Direct oxygen sensing via luminescence quenching	PpyPt NPs, PtTFPP/PtOEP, Rhenium-diimine complex, Ir-BTPHSA complex, Ir-PVP, RHyLI, PtG4	PLI, CLI	Real-time Quantitative Available to detect cyclic hypoxia	Poor biocompatibility Low penetration depth Visible/NIR-I only	NIR-II probes Upconversion nanoparticles	13,14,15,72,73,74,85
Biological	Enzyme-activated (NTRs, AzoRs) or Receptor-targeted (CAIX)	HDSF, X4, CNO, 3-azo-conjugated BODIPY, Hypoxia-targeted radiotracers (^18^F-FMISO,^18^F-FAZA,^18^F-HX4), CAIX-800, ^99m^Tc-PHC-102	FMI, PAI, PET/CT, SPECT	High specificity Good stability Easy accessibility	Off-target activation Limited sensitivity	Multimodal strategies	16,17,18,19,38,42,43,68,69,70,71,106
Chemical	Detection of hypoxia-relevant chemical compounds (pH, H_2_O_2_, H_2_S)	Ir-D, Au@Pt-Se NPs, CD-950, MB-m-borate, QN-Naph, DNNC, AGNPs, Ir-NP, SiRho-SHD-NTR, Ir-BTPHSA	FMI, PAI, PLI	High sensitivity Good specificity High SNR Real-time and rapid response	Cross-reactivity Complex synthesis Low penetration depth	Dual-lock probes Ratiometric imaging Self-calibrated probe platforms	24,25,26,27,29,30,31,32,67,72

NIR: near-infrared; CAIX: carbonic anhydrase IX; NTRs: nitroreductases; AzoRs: azoreductases; PLI: phosphorescence lifetime imaging; CLI: Cherenkov luminescence imaging; PAI: photoacoustic imaging; FMI: fluorescent molecular imaging; H_2_O_2_: hydrogen peroxide; H_2_S: hydrogen sulfide; ^18^F-FMISO: ^18^F-Fluoromisonidazole; ^18^F-FAZA: ^18^F-fluoroazomycin arabinoside; ^18^F-HX4: ^18^F-flortanidazole; PET/CT: positron emission tomography/computed tomography; SPECT: single photon emission computed tomography; SNR: signal-to-background ratio.

**Table 2:** Advantages and limitations of hypoxia imaging modalities.

Techniques	Labels	Signal measured	Advantages	Limitations	Cost	Throughput	Resolution	References
PET	Radiotracers	Positrons from radionuclides	High sensitivity and specificity	Radioactive Limited spatial resolution Low SNR	High	Low	5‒7 mm	33-48
BOLD-MRI	Label-free	Magnetic field alterations	Noninvasive Label-free High spatial resolution	Low sensitivity and specificity Susceptible to physiological factors Time-consuming scans	High	Low	1‒2 mm	50-54
OE-MRI	Label-free	Magnetic field alterations	Noninvasive Label-free High spatial resolution	Low sensitivity Limited specificity Time-consuming scans	High	Low	1‒2 mm	54-59
DCE-MRI	Gadolinium-based contrast agents	Vascular perfusion/permeability	High spatial resolution	Low sensitivity and specificity Time-consuming scans Require multiparametric analysis	High	Low	1‒2 mm	60-62
FMI	Fluorescent probes	Light	No radiation Low cost Live monitoring High sensitivity	Invasiveness, Limited tissue penetration	Low	High	2‒3 mm	16-19,24,25,27-32,63,64,71,106
PLI	Oxygen-sensitive luminescent probes	Light	No radiation, Direct oxygen levels quantification	Invasiveness Probe safety Complex imaging system Limited tissue penetration	Low	High	3‒5 mm	13-15,72-74
PAI	Label-free or Probes	Sound	Noninvasive High spatial-temporal resolutions Deep tissue penetration	Probe safety	Low	High	1 mm	26,67,75-82
CLI	Label-free or Oxyphros probes	Cherenkov photons	Live monitoring High sensitivity	Radioactive Limited tissue penetration	Low	High	3‒5 mm	83-87

PET: positron emission tomography; BOLD-MRI: blood oxygen level-dependent magnetic resonance imaging; OE-MRI: oxygen-enhanced magnetic resonance imaging; DCE-MRI: dynamic contrast enhancement magnetic resonance imaging; FMI: fluorescent molecular imaging; PLI: phosphorescence lifetime imaging; PAI: photoacoustic imaging; CLI: Cherenkov luminescence imaging.

**Table 3:** Clinical trials of hypoxia imaging in HNC.

Study Number	Outcome Measures	Imaging Modality	Tracer	Status	Outcomes	Last Update
NCT03646747	Feasibility of OE-MRI in detecting tumor hypoxia	OE-MRI	Label-free	Unknown Status	NA	2018
NCT04724096	Feasibility of OE-MRI in detecting tumor hypoxia	OE-MRI	Label-free	Completed	OE-MRI was feasible and well-tolerated in detecting hypoxia 113.	2023
NCT01829646	Prognostic value of functional MRI in detecting tumor hypoxia	DCE-MRI and DWI-MRI	Label-free	Recruiting	NA	2023
NCT03510390	Correlation between hypoxic volume and treatment response	BOLD and DWI	Label-free	Completed	NA	2021
NCT06041555	Feasibility of MRI in detecting tumor hypoxia	multiparametric MR	Label-free	Recruiting	NA	2024
NCT05246475	Voxel-level correlation of hypoxia MRI and PET estimates in detecting hypoxia	multiparametric MR and PET	^18^F-EF5	Recruiting	NA	2022
NCT06108089	Voxel-level correlation of hypoxia MRI and PET estimates in detecting hypoxia	multiparametric MR and PET	^18^F-FMISO	Recruiting	NA	2024
NCT05996432	Voxel-level correlation of hypoxia MRI and PET estimates in detecting hypoxia	multiparametric MR and PET	^18^F-FMISO	Recruiting	NA	2024
NCT04846309	Evaluating the safety and efficiency in hypoxia-guided dose-escalated radiotherapy	multiparametric MR and PET	^18^F-FMISO	Recruiting	NA	2025
NCT02352792	Prognostic value and relevant toxicity for hypoxia-guided dose-escalated radiotherapy	PET/CT	18F-FMISO	Completed	No relevant toxicities were detected; the dose escalation group experienced a local control benefit 90,111.	2015
NCT01212354	Evaluation of 2-year local control in hypoxia-guided dose-escalated radiotherapy	PET/CT	18F-FMISO	Recruiting	The dose escalation group experienced a 15% local control benefit 92.	2016
NCT01235052	Correlation between hypoxic volume and treatment response	PET/CT	^18^F-FMISO	Completed	NA	2017
NCT00180180	Correlation of hypoxia between primary tumors and lymph node metastases (LNM)	PET/CT	^18^F-FMISO	Completed	A significant correlation between FMISO-based hypoxia was observed in the primary tumor and large LNMs 103.	2020
NCT00606294	Evaluating the safety and efficiency in hypoxia-guided treatment optimization	PET/CT	^18^F-FMISO	Completed	No adverse events were observed; all patients are in the follow-up stage.	2023
NCT00606294	Correlation between hypoxic volume and distant metastasis (DM)	PET/CT	18F-FMISO	Completed	Persistent hypoxia was associated with increased DM risk and worse OS 104.	2023
NCT04995185	Evaluating the feasibility and efficiency of hypoxia-guided dose-escalated radiotherapy	PET/CT	18F-FMISO	Completed	NA	2024
NCT05348486	Evaluating the feasibility and efficiency of hypoxia-guided dose-escalated radiotherapy	PET/CT	18F-FMISO	Recruiting	NA	2025
NCT05544136	Evaluation of overall survival (OS) in hypoxia-guided dose-escalated radiotherapy	PET/CT	^18^F-FMISO	Recruiting	NA	2025
NCT06087614	Prognostic value and relevant toxicity for hypoxia-guided dose-escalated radiotherapy	PET/CT	18F-FMISO	Recruiting	NA	2025
NCT02207439	Correlation between hypoxic volume and treatment response	PET/CT	18F-FMISO or 18F-EF5	Completed	NA	2023
NCT01017224	Correlation between baseline hypoxia and treatment response	PET/CT	18F-FAZA	Completed	The TMR ≥1.6 at baseline was markedly associated with treatment failure 45.	2012
NCT02976051	Evaluating the efficiency of hypoxia-guided dose-escalated radiotherapy	PET/CT	18F-FAZA	Completed	The dose escalation group achieved a local control benefit of 20% 95.	2020
NCT01075399	Evaluating the repeatability of all parameters in hypoxia PET imaging	PET/CT	18F-HX4	Completed	All parameters were significantly correlated between scans 46.	2013
NCT01347281	Prognostic value for dynamic PET imaging of tumor hypoxia	PET/CT	18F-HX4	Completed	Dynamic changes of hypoxia correlated with prognostic outcome 93.	2017
NCT02976883	Correlation between hypoxic volume and treatment response	PET/CT	18F-HX4	Completed	NA	2019
NCT03003637	Correlation between hypoxic volume and treatment response	PET/CT	^18^F-HX4	Completed	An on-treatment decrease in hypoxia signature was observed in responders to immunotherapy 97.	2023
NCT06716892	Evaluating the potential of preoperative hypoxia detection in HNC	PAI	Label-free	Recruiting	NA	2024

HNC: head and cancer; PET: positron emission tomography; CT: computed tomography; MR: magnetic resonance; MRI: magnetic resonance imaging; OE-MRI: oxygen-enhanced magnetic resonance imaging; DCE: dynamic contrast-enhanced magnetic resonance imaging; DWI-MRI: diffusion-weighted magnetic resonance imaging; PAI: photoacoustic imaging; NA: not available.

## Abbreviations

HNC: head and neck cancer
PET: positron emission tomography
MRI: magnetic resonance imaging
O_2_: oxygen
CT: computed tomography
^18^F-FAZA: [^18^F] fluoroazomycin arabinoside
^18^F-HX4: [^18^F] flortanidazole
^18^F-FMISO: [^18^F] fluoromisonidazole
OMI: optical molecular imaging
HAPs: hypoxia-activated prodrugs
*p*O_2_: partial oxygen pressure
*_S_*O_2_: oxygen saturation
CAIX: carbonic anhydrase IX
NTRs: nitroreductases
AzoRs: azoreductases
H_2_O_2_: hydrogen peroxide
H_2_S: hydrogen sulfide
HIF-1α: hypoxia-inducible factor-1α
SNR: signal-to-noise ratio
TBR: tumor-background ratio
BOLD-MRI: blood oxygen level-dependent MRI
OE-MRI: oxygen-enhanced MRI
DCE-MRI: dynamic contrast-enhanced MRI
OMI: optical molecular imaging
ACQ: aggregation-caused quenching
FRET: fluorescence resonance energy transfer
PLI: phosphorescence lifetime imaging
PAI: photoacoustic imaging
OE-OT: oxygen-enhanced optoacoustic tomography
DCE-OT: dynamic contrast-enhanced optoacoustic tomography
CLI: Cherenkov luminescence imaging
PD-L1: programmed cell death ligand 1
AI: artificial intelligence
